# Investigation of *TCF7L2, LEP* and *LEPR* polymorphisms with esophageal squamous cell carcinomas

**DOI:** 10.18632/oncotarget.22619

**Published:** 2017-11-17

**Authors:** Hao Qiu, Xunting Lin, Weifeng Tang, Chao Liu, Yu Chen, Hao Ding, Mingqiang Kang, Shuchen Chen

**Affiliations:** ^1^ Department of Immunology, School of Medicine, Jiangsu University, Zhenjiang, Jiangsu Province, China; ^2^ Department of Gastroenterology, Zhongshan Hospital Xiamen University, Xiamen, Fujian Province, China; ^3^ Department of Cardiothoracic Surgery, Affiliated People’s Hospital of Jiangsu University, Zhenjiang, Jiangsu Province, China; ^4^ Department of Medical Oncology, Fujian Cancer Hospital, Fujian Medical University Cancer Hospital, Fuzhou, Fujian Province, China; ^5^ Department of Respiratory Disease, Affiliated People’s Hospital of Jiangsu University, Zhenjiang, Jiangsu Province, China; ^6^ Department of Thoracic Surgery, Fujian Medical University Union Hospital, Fuzhou, Fujian Province, China; ^7^ Key Laboratory of Ministry of Education for Gastrointestinal Cancer, Fujian Medical University, Fuzhou, Fujian Province, China; ^8^ Fujian Key Laboratory of Tumor Microbiology, Fujian Medical University, Fuzhou, Fujian Province, China

**Keywords:** TCF7L2, LEP, LEPR, polymorphism, ESCC

## Abstract

Single nucleotide polymorphisms (SNPs) in energy metabolism related gene may be key agents in the development of human malignancies. In this study, we aimed to examine the association of *transcription factor 7-like 2*, *Leptin* (*LEP*) and *LEP receptor* (*LEPR*) polymorphisms with esophageal squamous cell carcinoma (ESCC). A total of 507 ESCC cases and 1,496 controls were enrolled. We found that *LEPR* rs6588147 AA genotype was associated with ESCC risk (AA vs. GG/GA: adjusted OR=1.90, 95%CI=1.00–3.61, *P*=0.049). In the stratified analyses, *LEPR* rs6588147 G>A polymorphism increased the risk of ESCC (<63 years subgroup: AA vs. GG: adjusted OR=2.58, 95%CI=1.00–6.62, *P*=0.049 and AA vs. GA/GG: adjusted OR=2.71, 95%CI=1.06–6.91, *P*=0.038; male subgroup: AA vs. GG: adjusted OR=2.19, 95%CI=1.02–4.67, *P*=0.044 and AA vs. GA/GG: adjusted OR=2.26, 95%CI=1.06–4.80, *P*=0.035). However, *LEP* rs7799039 A>G decreased the risk of ESCC (≥63 years subgroup: GG vs. AA: adjusted OR=0.47, 95%CI=0.23–0.95, *P*=0.035 and GG vs. AA/AG: adjusted OR=0.48, 95%CI=0.24–0.96, *P*=0.038; BMI≥24 kg/m^2^ subgroup: AG vs. AA: adjusted OR=0.66, 95%CI=0.45–0.99, *P*=0.044). In addition, *LEPR* rs1137101 G>A polymorphism decreased ESCC risk in some subgroups (ever smoking subgroup: GA vs. GG: adjusted OR=0.66, 95%CI=0.44–1.00, *P*=0.049; ever drinking subgroup: GA vs. GG: adjusted OR=0.54, 95%CI=0.31–0.95, *P*=0.031 and GA/AA vs. GG: adjusted OR=0.54, 95%CI=0.31–0.93, *P*=0.027). Our findings suggest that *LEPR* rs6588147 G>A polymorphism is associated with the increased risk of ESCC; however, *LEP* rs7799039 A>G and *LEPR* rs1137101 G>A polymorphisms may be protective factors for ESCC.

## INTRODUCTION

In China, esophageal cancer (EC) is the fourth most commonly diagnosed cancer in males and the fifth in females, with an estimated 477,900 new patients and 375,000 related deaths occurring in 2015 [1]. Esophageal squamous cell carcinoma (ESCC) is the main form of EC in China and Eastern Asia. The contributing risk factors for ESCC are not fully known, but are thought to involve low intake of vegetables and fruits, poor nutritional status, smoking and eating and/or drinking at high temperatures. However, these primarily identified risk factors could not account for all the etiology of ESCC. Nowadays, there are convincing evidences that obesity increases the susceptibility of many malignancies, including EC, postmenopausal breast cancer, endometrial cancer, colorectal cancer, pancreatic cancer and liver cancer [2]. A recent study indicated that preoperative metabolic syndrome might be an effective predictor of ESCC mortality [3]. These accumulating evidences suggested that obesity and diabetes related gene might play vital roles in the development of EC.

The *transcription factor 7-like 2* (*TCF7L2*) gene maps to the long arm of chromosome 10q25.3. TCF7L2 belongs to the high mobility group-box (HMGB) family [4] and is a versatile transcription factor. The TCF7L2 protein regulates Wnt/β-catenin signaling pathway [5], therefore it plays important roles in the development and growth of various cells [6, 7]. Ishiguro *et al.* reported that TCF7L2 expression was associated with a poor prognosis of ESCC [8]. A previous study suggested that *TCF7L2* rs7903146 locus might exert its enhancer function by interacting with HMGB1 [9]. *TCF7L2* single-nucleotide polymorphisms (SNPs) are proposed susceptibility factors for the development of cancer. Previous studies indicated that *TCF7L2* rs7903146 (C/T) polymorphism might influence the risk of breast cancer [10, 11]. *TCF7L2* rs290481 T>C polymorphism located on near the 3’ end of this gene. Ling *et al.* reported that this SNP was associated with hepatocellular carcinoma susceptibility with marginal significance [12]. However, the association between these *TCF7L2* SNPs and ESCC risk was not explored.

The *Leptin* (*LEP*) gene maps to chromosome 7q31.3. LEP is secreted by white adipose tissue and has been identified to be involved in endocrinologic metabolism [13]. It is thought that LEP may regulate the activation and serum levels of insulin. Thus, LEP may involve in the etiology of obesity [14], type 2 diabetes (T2DM) [15] and pathophysiology of carcinoma [16, 17]. LEP receptor (LEPR, also known as CD295) is a single transmembrane protein in human and distributes in various tissues [18]. LEP combines to LEPR and exerts its important roles in the development of metabolic disorders and malignancies. Several studies demonstrated that the elevated LEP levels might affect the onset and progression of many malignancies [19–22]. Thus, LEP and LEPR may be correlated with the development of ESCC. Results of meta-analyses found that both rs7799039 A>G and rs2167270 G>A polymorphisms in *LEP* gene might influence the risk of cancer [23–25]. In addition, a case-control study found that *LEP* rs2167270 G>A was associated with the risk of esophageal adenocarcinoma [26]. There are several explanations for the function of these two *LEP* polymorphisms. It is suggested that rs7799039 A>G polymorphism in the upstream region of LEP gene can affect leptin expression, possibly at the transcriptional level, thereby altering adipose secretion levels of the hormone [27]. Additionally, *LEP* rs2167270 G>A is a 5’-utr SNP and may play regulatory roles in translation and stability of mRNA. LEPR rs1137100 G>A, rs1137101 G>A polymorphisms are missense SNPs and may alter the structure and the function of LEPR protein. Doecke *et al.* found *LEPR* rs1137100 G>A, rs1137101 G>A polymorphisms influence the risk of esophageal adenocarcinoma in Caucasians [26]. *LEPR* rs6588147 G>A polymorphism locates on the intron region of LEPR gene. Slattery *et al.* found that *LEPR* rs6588147 G>A polymorphism affected risk of colon cancer among men [28]. However, the association between *LEPR* rs1137100 G>A, rs1137101 G>A and rs6588147 G>A polymorphisms and ESCC risk remains unknown in Asians.

In this case-control study, we aimed to examine the potential association of *TCF7L2*, *LEP* and *LEPR* polymorphisms with the risk of ESCC in Eastern Chinese Han populations. The *TCF7L2* rs7903146 C>T, rs290481 T>C, *LEP* rs7799039 A>G, rs2167270 G>A and *LEPR* rs1137100 G>A, rs1137101 G>A and rs6588147 G>A polymorphisms were genotyped by SNPscan genotyping assays in 507 ESCC cases and 1,496 non-cancer controls.

## RESULTS

### Baseline characteristics

There were 2,003 participants in the present case-control study including 507 ESCC patients (377 males and 130 females) and 1,496 non-cancer controls (1,084 males and 412 females). The age and sex were well matched in two groups (P = 0.994, P = 0.406, respectively, Table 1). The mean ± SD of weight and body mass index (BMI) was significantly higher in controls compared with ESCC patients (*P* < 0.05). However, the mean ± SD of height was not significant (*P* > 0.05). The proportion of smoking and drinking was significantly higher in ESCC patients compared with controls (*P* < 0.05). Locus information of *TCF7L2*, *LEP* and *LEPR* polymorphisms is listed in Table 2. The genotyping success rates for *TCF7L2* rs7903146C>T, rs290481 T>C, *LEP* rs7799039 A>G, rs2167270 G>A and *LEPR* rs1137100 G>A, rs1137101 G>A and rs6588147 G>A SNPs were 99.50%,99.45%, 99.50%, 99.40%, 99.50%, 99.50% and 99.50%, respectively. Minor allele frequency (MAF) in controls is listed in Table 2, which is very similar to the data of Chinese population. In addition, the distributions of the *TCF7L2* rs7903146C>T, rs290481 T>C, *LEP* rs7799039 A>G, rs2167270 G>A and *LEPR* rs1137100 G>A, rs1137101 G>A and rs6588147 G>A genotypes in controls conform to Hardy-Weinberg equilibrium (HWE).

**Table 1:** Distribution of selected demographic variables and risk factors in ESCC cases and controls

Variable	Cases (n=507)		Controls (n=1, 496)		*P* ^a^
	n	%	n	%	
Age (years)	62.77 (±8.01)		62.77 (±8.84)		0.994
Age (years)					0.225
< 63	271	53.45	753	50.33	
≥ 63	236	46.55	743	49.67	
Sex					0.406
Male	377	74.36	1,084	72.46	
Female	130	25.64	412	27.54	
Tobacco use					<0.001
Never	247	48.72	1,090	72.86	
Ever	260	51.28	406	27.14	
Alcohol use					<0.001
Never	341	67.26	1,329	88.84	
Ever	166	32.74	167	11.16	
Height (cm)	166.0 (±7.29)		166.1 (±7.08)		0.743
Weight (kg)	61.54 (±9.83)		66.11 (±9.92)		<0.001
BMI (kg/m^2^)	22.27 (±2.90)		23.91 (±3.03)		<0.001
BMI (kg/m^2^)					<0.001
< 24	370		779		
≥ 24	137		717		

^a^ Two-sided *χ*^2^ test and student t test; Bold values are statistically significant (*P* <0.05). BMI: body mass index.

**Table 2:** Primary information for *TCF7L2* rs7903146C>T, rs290481 T>C, *LEP* rs7799039 A>G, rs2167270 G>A and *LEPR* rs1137100 G>A, rs1137101 G>A and rs6588147 G>A polymorphisms

Genotyped SNPs	Chromosome	Chr Pos (NCBI Build 37)	Region	MAF^a^ for Chinese in database	MAF in our controls (n = 1, 496)	*P* value for HWE^b^ test in our controls	Genotyping method	Genotyping value (%)
*TCF7L2* rs7903146 C>T	10	114758349	Intron 4	0.026	0.031	0.733	SNPscan	99.50
*TCF7L2* rs290481 T>C	10	114923825	Intron 13	0.405	0.387	0.097	SNPscan	99.45
*LEP* rs7799039 A>G	7	127878783	Promoter	0.201	0.266	0.543	SNPscan	99.50
*LEP* rs2167270 G>A	7	127881349	5’ UTR	0.175	0.222	0.324	SNPscan	99.40
*LEPR* rs1137100 G>A	1	66036441	Exon 4	0.169	0.160	0.316	SNPscan	99.50
*LEPR* rs1137101 G>A	1	66058513	Exon 6	0.111	0.122	0.763	SNPscan	99.50
*LEPR* rs6588147 G>A	1	65935494	Intron 2	0.150	0.150	0.260	SNPscan	99.50

^a^ MAF: minor allele frequency.

^b^ HWE: Hardy–Weinberg equilibrium.

### Association of TCF7L2 rs7903146C>T, rs290481 T>C, LEP rs7799039 A>G, rs2167270 G>A and LEPR rs1137100 G>A, rs1137101 G>A and rs6588147 G>A polymorphisms with ESCC risk

The genotype distributions of *TCF7L2* rs7903146C>T, rs290481 T>C, *LEP* rs7799039 A>G, rs2167270 G>A and *LEPR* rs1137100 G>A, rs1137101 G>A and rs6588147 G>A polymorphisms are listed in Table 3. In the analysis of *LEPR* rs6588147 G>A polymorphism, we found significant differences in the distribution of the rs6588147 AA genotype compared with the rs6588147 GG genotype and rs6588147 AA genotype compared with the rs6588147 GA/GG genotypes between 507 ESCC cases and 1,496 controls [AA vs. GG: crude odds ratio (OR) = 1.87, 95% confidence interval (CI) = 1.02–3.43, *P* = 0.042 and AA vs. GG/GA: crude OR = 1.93, 95% CI = 1.06–3.53, *P* = 0.031 (Table 3)]. Results of multivariate linear regression analysis indicated that *LEPR* rs6588147 G>A polymorphism increased the risk of ESCC. When the *LEPR* rs6588147 GG/GA genotypes were used as the reference group, the *LEPR* rs6588147 AA genotype was associated with the increased risk of ESCC [AA vs. GG/GA: adjusted OR = 1.90, 95% CI = 1.00–3.61, *P* = 0.049 (Table 3)]. However, we found that *TCF7L2* rs7903146C>T, rs290481 T>C, *LEP* rs7799039 A>G, rs2167270 G>A and *LEPR* rs1137100 G>A, rs1137101 G>A polymorphisms were not associated with the development of overall ESCC (Table 3).

**Table 3:** Logistic regression analyses of association between *TCF7L2* rs7903146C>T, rs290481 T>C, *LEP* rs7799039 A>G, rs2167270 G>A and *LEPR* rs1137100 G>A, rs1137101 G>A and rs6588147 G>A polymorphisms and risk of ESCC

Genotype	ESCC cases (n=507)		Controls (n=1, 496)		Crude OR (95%CI)	*P*	Adjusted OR ^a^ (95%CI)	*P*
	n	%	n	%				
*TCF7L2* rs7903146C>T								
CC	475	94.25	1,399	93.96	1.00			
CT	29	5.75	89	5.98	0.96(0.62-1.48)	0.847	1.03(0.65-1.62)	0.908
TT	0	0	1	0.07	-	-	-	-
CT+TT	29	5.75	90	6.04	0.95(0.62-1.46)	0.814	1.01(0.64-1.60)	0.954
CC+CT	504	100.00	1488	99.93	1.00		1.00	
TT	0	0	1	0.07	-	-	-	-
T allele	29	2.88	91	3.06				
*TCF7L2* rs290481 T>C								
TT	195	38.77	575	38.62	1.00			
TC	228	45.33	676	45.40	0.99(0.79-1.23)	0.903	0.96(0.76-1.22)	0.748
CC	80	15.90	238	15.98	0.98(0.73-1.33)	0.911	0.99(0.71-1.36)	0.927
TC+CC	308	61.23	914	61.38	0.99(0.81-1.22)	0.952	0.98(0.78-1.22)	0.830
TT+TC	423	84.10	1,251	84.02	1.00		1.00	
CC	80	15.90	238	15.98	0.99(0.75-1.31)	0.967	1.01(0.75-1.36)	0.949
C allele	388	38.57	1,152	38.68				
*LEP* rs7799039 A>G								
AA	291	57.74	797	53.53	1.00		1.00	
AG	184	36.51	591	39.69	0.85(0.69-1.05)	0.138	0.85(0.67-1.06)	0.144
GG	29	5.75	101	6.78	0.79(0.51-1.21)	0.275	0.73(0.46-1.17)	0.191
AG+GG	213	42.26	692	46.47	0.84(0.69-1.03)	0.101	0.83(0.67-1.03)	0.091
AA+AG	475	94.25	1,388	93.22	1.00		1.00	
GG	29	5.75	101	6.78	0.84(0.55-1.28)	0.419	0.79(0.50-1.24)	0.300
G allele	242	24.01	793	26.63				
*LEP* rs2167270 G>A								
GG	318	63.35	894	60.04	1.00		1.00	
GA	165	32.87	528	35.46	0.87(0.70-1.08)	0.213	0.87(0.69-1.09)	0.220
AA	19	3.78	67	4.50	0.79(0.47-1.34)	0.382	0.81(0.47-1.42)	0.469
GA+AA	184	36.65	595	39.96	0.87(0.71-1.07)	0.190	0.86(0.69-1.08)	0.198
GG+GA	483	96.22	1,422	95.50	1.00		1.00	
AA	19	3.78	67	4.50	0.84(0.50-1.40)	0.496	0.86(0.49-1.50)	0.591
A allele	203	20.22	662	22.23				
*LEPR* rs1137100 G>A								
GG	342	67.86	1,045	70.18	1.00		1.00	
GA	147	29.17	411	27.60	1.09(0.87-1.37)	0.448	1.08(0.85-1.38)	0.517
AA	15	2.98	33	2.22	1.39(0.74-2.58)	0.304	1.30(0.67-2.52)	0.436
GA+AA	162	32.14	444	29.82	1.12(0.90-1.39)	0.327	1.10(0.87-1.39)	0.417
GG+GA	489	97.02	1,456	97.78	1.00		1.00	
AA	15	2.98	33	2.22	1.35(0.73-2.51)	0.338	1.27(0.66-2.46)	0.472
A allele	177	17.56	477	16.02				
*LEPR* rs1137101 G>A								
GG	390	77.38	1,146	76.96	1.00		1.00	
GA	108	21.43	322	21.63	0.98(0.77-1.26)	0.898	0.91(0.70-1.18)	0.473
AA	6	1.19	21	1.41	0.84(0.34-2.09)	0.705	0.91(0.35-2.37)	0.848
GA+AA	114	22.62	343	23.04	0.98(0.77-1.24)	0.848	0.91(0.70-1.18)	0.468
GG+GA	498	98.81	1,468	98.59	1.00		1.00	
AA	6	1.19	21	1.41	0.84(0.34-2.10)	0.712	0.93(0.36-2.42)	0.884
A allele	120	11.90	364	12.22				
*LEPR* rs6588147 G>A								
GG	367	72.82	1,070	71.86	1.00		1.00	
GA	119	23.61	391	26.26	0.89(0.70-1.12)	0.316	0.85(0.66-1.09)	0.199
AA	18	3.57	28	1.88	**1.87(1.02-3.43)**	**0.042**	1.82(0.96-3.46)	0.068
GA + AA	137	27.18	419	28.14	0.95(0.76-1.20)	0.680	0.91(0.72-1.16)	0.465
GG+GA	486	96.43	1,461	98.12	1.00		1.00	
AA	18	3.57	28	1.88	**1.93(1.06-3.53)**	**0.031**	**1.90(1.00-3.61)**	**0.049**
A allele	155	15.38	447	15.01				

^a^ Adjusted for age, sex, BMI, alcohol use and smoking status.

Bold values are statistically significant (*P* <0.05).

### Association of TCF7L2 rs7903146C>T, rs290481 T>C, LEP rs7799039 A>G, rs2167270 G>A and LEPR rs1137100 G>A, rs1137101 G>A and rs6588147 G>A polymorphisms with ESCC risk in Different Stratification Groups

Table 4 shows the genotype frequencies of *LEP* rs7799039 A>G polymorphism in the subgroup analyses. In ≥63 years subgroup, after adjustment for gender, smoking status, BMI and alcohol use, the *LEP* rs7799039 GG genotype decreased ESCC risk compared with the *LEP* rs7799039 AA genotype genotype or *LEP* rs7799039 AA/AG [GG vs. AA: adjusted OR = 0.47, 95% CI 0.23–0.95, *P* = 0.035 and GG vs. AA/AG: adjusted OR = 0.48, 95% CI = 0.24–0.96, *P* = 0.038 (Table 4)]. In BMI ≥ 24 kg/m^2^ subgroup, after adjustment for age, gender, smoking status and alcohol use, we found that *LEP* rs7799039 AG genotype decreased the risk of ESCC [AG vs. AA: adjusted OR = 0.66, 95% CI 0.45–0.99, *P* = 0.044 (Table 4)].

**Table 4:** Stratified analyses between *LEP* rs7799039 A>G polymorphism and ESCC risk by sex, age, BMI, smoking status and alcohol consumption

Variable	*LEP* rs7799039 A>G (case/control)^a^			Adjusted OR^b^ (95% CI); *P*				
	AA	AG	GG	AA	AG	GG	AG/GG	GG vs. (AG/AA)
Sex								
Male	222/581	134/425	19/72	1.00	0.81(0.62-1.06); *P*: 0.117	0.60(0.33-1.06); *P*: 0.079	0.77(0.60-1.00); *P*: 0.052	0.65(0.37-1.15); *P*: 0.136
Female	69/216	50/166	10/29	1.00	0.97(0.63-1.50); *P*: 0.901	1.324 (0.60-2.97); *P*: 0.475	1.03(0.68-1.55); *P*: 0.897	1.36 (0.62-2.95); *P*: 0.442
Age								
<63	139/395	79/306	18/46	1.00	0.78(0.56-1.11); *P*: 0.166	1.31 (0.69-2.50); *P*: 0.409	0.84(0.60-1.16); *P*: 0.282	1.43 (0.76-2.69); *P*: 0.263
≥63	152/402	105/285	11/55	1.00	0.95(0.70-1.29); *P*: 0.737	**0.47(0.23-0.95);** ***P*: 0.035**	0.88 (0.65-1.18); *P*: 0.395	**0.48 (0.24-0.96);** ***P*: 0.038**
Smoking status								
Never	146/589	83/427	16/70	1.00	0.79(0.59-1.08); *P*: 0.135	0.99(0.55-1.78); *P*: 0.970	0.83(0.62-1.10); *P*: 0.190	1.09(0.61-1.93); *P*: 0.779
Ever	145/208	101/164	13/31	1.00	0.92(0.65-1.31); *P*: 0.637	0.49(0.23-1.02); *P*: 0.057	0.84(0.60-1.18); *P*: 0.306	0.50(0.24-1.04); *P*: 0.063
Alcohol consumption								
Never	198/706	122/526	18/91	1.00	0.82(0.63-1.06); *P*: 0.135	0.72(0.42-1.23); *P*: 0.229	0.81 (0.63-1.04); *P*: 0.097	0.78(0.46-1.33); *P*: 0.359
Ever	93/91	62/65	11/10	1.00	1.06(0.64-1.77); *P*: 0.820	0.75(0.28-1.96); *P*: 0.552	0.99(0.61-1.60); *P*: 0.955	0.72(0.28-1.85); *P*: 0.492
BMI (kg/m^2^)								
<24	210/436	137/285	20/53	1.00	0.96(0.72-1.26); *P*: 0.744	0.63(0.35-1.13); *P*: 0.118	0.90 (0.69-1.18); *P*: 0.458	0.64(0.36-1.13); *P*: 0.126
≥24	81/361	47/306	9/48	1.00	**0.66(0.45-0.99);** ***P*: 0.044**	0.93(0.43-1.99); *P*: 0.847	0.69(0.48-1.01); *P*: 0.058	1.09(0.52-2.31); *P*: 0.816

^a^ For *LEP* rs7799039 A>G, the genotyping was successful in 507 (99.41%) ESCC cases, and 1,496 (99.53%) controls.

^b^ Adjusted for multiple comparisons [age, sex, BMI, smoking status and alcohol consumption (besides stratified factors accordingly)] in a logistic regression model.

The genotype frequencies of *LEPR* rs1137101 G>A polymorphism in the subgroup analyses are showed in Table 5. In ever smoking subgroup, after adjustment for gender, age, BMI and alcohol use, the *LEPR* rs1137101 GA genotype was associated with the decreased risk of ESCC [GA vs. GG: adjusted OR = 0.66, 95% CI 0.44–1.00, *P* = 0.049 (Table 5)]. In ever drinking subgroup, after adjustment for gender, smoking status, BMI and age, we found that *LEPR* rs1137101 GA and GA/AA genotypes decreased the risk of ESCC [GA vs. GG: adjusted OR = 0.54, 95% CI 0.31–0.95, *P* = 0.031 and GA/AA vs. GG: adjusted OR = 0.54, 95% CI 0.31–0.93, *P* = 0.027 (Table 5)].

**Table 5:** Stratified analyses between *LEPR* rs1137101 G>A polymorphism and ESCC risk by sex, age, BMI, smoking status and alcohol consumption

Variable	*LEPR* rs1137101 G>A (case/control)^a^			Adjusted OR^b^ (95% CI); *P*				
	GG	GA	AA	GG	GA	AA	GA/AA	AA vs. (GA/GG)
Sex								
Male	292/832	78/235	5/11	1.00	0.84(0.61-1.15); *P*: 0.275	1.52(0.49-4.75); *P*: 0.473	0.87(0.64-1.18); *P*: 0.353	1.57(0.50-4.91); *P*: 0.435
Female	98/314	30/87	1/10	1.00	1.11(0.68-1.81); *P*: 0.686	0.27(0.03-2.24); *P*: 0.226	1.02(0.63-1.65); *P*: 0.943	0.27 (0.03-2.20); *P*: 0.220
Age								
<63	177/579	55/157	4/11	1.00	1.06 (0.72-1.57); *P*: 0.772	1.77 (0.51-6.13); *P*: 0.370	1.09(0.74-1.59); *P*: 0.666	1.73(0.50-5.98); *P*: 0.387
≥63	213/567	53/165	2/10	1.00	0.75(0.52-1.08); *P*: 0.123	0.43(0.09-2.02); *P*: 0.283	0.74(0.52-1.06); *P*: 0.097	0.46(0.10-2.16); *P*: 0.323
Smoking status								
Never	186/848	56/221	3/17	1.00	1.15(0.82-1.61); *P*: 0.432	0.72(0.20-2.56); *P*: 0.613	1.12(0.80-1.57); *P*: 0.504	0.70(0.20-2.49); *P*: 0.585
Ever	204/298	52/101	3/4	1.00	**0.66(0.44-1.00);** ***P*: 0.049**	1.51(0.30-7.58); *P*: 0.616	0.68(0.46-1.02); *P*: 0.063	1.65 (0.33-8.24); *P*: 0.543
Alcohol consumption								
Never	260/1,028	73/276	5/19	1.00	1.00(0.74-1.35); *P*: 0.999	1.04(0.37-2.89); *P*: 0.943	1.01(0.75-1.35); *P*: 0.953	1.04(0.38-2.89); *P*: 0.939
Ever	130/118	35/46	1/2	1.00	**0.54(0.31-0.95);** ***P*: 0.031**	0.56(0.04-8.70); *P*: 0.679	**0.54(0.31-0.93);** ***P*: 0.027**	0.64(0.04-9.68); *P*: 0.750
BMI (kg/m^2^)								
<24	279/600	83/165	5/9	1.00	0.99(0.72-1.36); *P*: 0.930	1.32(0.42-4.18); *P*: 0.633	1.01(0.74-1.38); *P*: 0.972	1.33(0.42-4.20); *P*: 0.623
≥24	111/546	25/157	1/12	1.00	0.76(0.47-1.22); *P*: 0.250	0.39(0.05-3.12); *P*: 0.376	0.73(0.45-1.16); *P*: 0.183	0.41(0.05-3.29); *P*: 0.405

^a^ For *LEPR* rs1137101 G>A, the genotyping was successful in 507 (99.41%) ESCC cases, and 1,496 (99.53%) controls.

^b^ Adjusted for multiple comparisons [age, sex, BMI, smoking status and alcohol consumption (besides stratified factors accordingly)] in a logistic regression model.

Table 6 shows the genotype frequencies of *LEPR* rs6588147 G>A polymorphism in the subgroup analyses. In <63 years subgroup, after adjustment for gender, smoking status, BMI and alcohol use, the *LEPR* rs6588147 AA genotype increased ESCC risk compared with the *LEPR* rs6588147 GG and GA/GG genotypes [AA vs. GG: adjusted OR = 2.58, 95% CI 1.00–6.62, *P* = 0.049 and AA vs. GA/GG: adjusted OR = 2.71, 95% CI 1.06–6.91, *P* = 0.038 (Table 6)]. In male subgroup, after adjustment for age, smoking status, BMI and alcohol use, the *LEPR* rs6588147 AA genotype was associated with the increased risk of ESCC [AA vs. GG: adjusted OR = 2.19, 95% CI 1.02–4.67, *P* = 0.044 and AA vs. GA/GG: adjusted OR = 2.26, 95% CI 1.06–4.80, *P* = 0.035 (Table 6)]. However, in ever drinking subgroup, after adjustment for age, gender, smoking status and BMI, the *LEPR* rs6588147 GA genotype decreased the risk of ESCC [GA vs. GG: adjusted OR = 0.54, 95% CI 0.31–0.92, *P* = 0.024 (Table 6)].

**Table 6:** Stratified analyses between *LEPR* rs6588147 G>A polymorphism and ESCC risk by sex, age, BMI, smoking status and alcohol consumption

Variable	*LEPR* rs6588147 G>A (case/control)^a^			Adjusted OR^b^ (95% CI); *P*				
	GG	GA	AA	GG	GA	AA	GA/AA	AA vs. (GA/GG)
Sex								
Male	267/769	94/290	14/19	1.00	0.89(0.67-1.20); *P*: 0.449	**2.19(1.02-4.67);** ***P*: 0.044**	0.97(0.73-1.29); *P*: 0.834	**2.26(1.06-4.80);** ***P*: 0.035**
Female	100/301	25/101	4/9	1.00	0.72(0.43-1.20); *P*: 0.204	1.19(0.34-4.22); *P*: 0.785	0.76(0.47-1.24); *P*: 0.274	1.29(0.37-4.55); *P*: 0.688
Age								
<63	168/527	59/206	9/14	1.00	0.80(0.55-1.16); *P*: 0.233	**2.58(1.00-6.62);** ***P*: 0.049**	0.88(0.62-1.26); *P*: 0.484	**2.71(1.06-6.91);** ***P*: 0.038**
≥63	199/543	60/185	9/14	1.00	0.84(0.59-1.20); *P*: 0.339	1.40(0.58-3.39); *P*: 0.458	0.90(0.64-1.26); *P*: 0.534	1.48(0.61-3.56); *P*: 0.386
Smoking status								
Never	180/787	56/279	9/20	1.00	0.89(0.63-1.24); *P*: 0.486	1.88(0.82-4.31); *P*: 0.139	0.96(0.70-1.32); *P*: 0.807	1.94(0.85-4.44); *P*: 0.117
Ever	187/283	63/112	9/8	1.00	0.80(0.54-1.17); *P*: 0.248	2.00(0.71-5.66); *P*: 0.191	0.86(0.59-1.25); *P*: 0.438	2.12 (0.75-5.97); *P*: 0.155
Alcohol consumption								
Never	245/961	80/335	13/27	1.00	0.92(0.69-1.23); *P*: 0.590	1.69(0.84-3.40); *P*: 0.145	0.99(0.75-1.30); *P*: 0.944	1.73(0.86-3.47); *P*: 0.124
Ever	122/109	39/56	5/1	1.00	**0.54(0.31-0.92);** ***P*: 0.024**	5.03(0.48-52.46); *P*: 0.177	0.60(0.35-1.01); *P*: 0.056	5.79(0.56-59.52); *P*: 0.139
BMI (kg/m^2^)								
<24	261/552	92/204	14/18	1.00	0.94(0.69-1.28); *P*: 0.700	1.79(0.84-3.82); *P*: 0.130	1.01(0.76-1.36); *P*: 0.936	1.83(0.86-3.89); *P*: 0.115
≥24	106/518	27/187	4/10	1.00	0.67(0.42-1.07); *P*: 0.093	1.96(0.59-6.59); *P*: 0.275	0.73(0.47-1.14); *P*: 0.168	2.14(0.64-7.17); *P*: 0.215

^a^ For *LEPR* rs1137101 G>A, the genotyping was successful in 507 (99.41%) ESCC cases, and 1,496 (99.53%) controls.

^b^ Adjusted for multiple comparisons [age, sex, BMI, smoking status and alcohol consumption (besides stratified factors accordingly)] in a logistic regression model.

In addition, after a logistic regression analysis, we found that *TCF7L2* rs7903146C>T, rs290481 T>C, *LEP* rs2167270 G>A and *LEPR* rs1137100 G>A polymorphisms were not associated with the risk of ESCC in any subgroup (data not shown).

## DISCUSSION

The pathogenesis of ESCC was very complex. Multiple factors (e.g. a number of genetic and environmental factors) may contribute to the etiology of ESCC. Understanding of the individual’s heredity background may be helpful for the prevention and treatment of ESCC. In this study, we selected energy metabolism and insulin-sensibility relative gene (*TCF7L2*, *LEP* and *LEPR*) polymorphisms and focused on their susceptibility to ESCC. The association between *LEPR* rs6588147 G>A polymorphism and the increased risk of overall ESCC was identified. We also found that *LEPR* rs6588147 G>A polymorphism increased the risk of ESCC in <63 years and male subgroups. *LEP* rs7799039 A>G was associated with the risk of ESCC in ≥63 years and BMI ≥ 24 kg/m^2^ subgroups. In addition, *LEPR* rs1137101 G>A polymorphism decreased the risk of ESCC in ever smoking and ever drinking subgroups.

There was a difference in the *LEPR* rs6588147 G>A polymorphism between overall ESCC patients and non-cancer controls. The *LEPR* rs6588147 AA genotype were higher in ESCC patients compared with controls, indicating that *LEPR* rs6588147 AA genotype may contribute to esophageal carcinogenesis. The *LEPR* rs6588147 G>A polymorphism is located on intron of *LEPR* gene. It may be difficult to interpret the exact function of intronic polymorphism. However, the possible interpretations may be as follows. The intronic polymorphism rs6588147 G>A is located near the regulatory components or splice acceptor site, where any slight variant may lead to the disruption of the splice site and induce aberrant splicing [29]. This SNP probably influences the expression of the LEPR protein by altering mRNA splicing. However, we found that *LEPR* rs6588147 AA genotype may decrease the risk of ESCC in ever drinking subgroup. These findings seemed to be controversial. The probable reason might be due to the limited sample size in this subgroup, which could generate an unauthentic results.

LEP is mainly secreted by adipose tissue, and has been suggested to promote tumor growth [30]. Some studies indicated that the serum LEP level was significantly higher in breast cancer patients compared with which in controls both pre-menopausal and post-menopausal [31, 32]. A number of studies have found that LEP may play vital roles in cell proliferation, apoptosis, cell migration and angiogenesis [33, 34]. Results of several meta-analyses suggested that *LEP* rs7799039 G allele might decrease the risk of multiple cancers [24, 25, 35–37]. However, there was only one study focused on the relationship between *LEP* rs7799039 A>G polymorphism and cancer risk in Asian populations. Thus, the association of this polymorphism with cancer risk might be unclear in Asians. In this study, we conducted a case-control study focused on the association between *LEP* rs7799039 A>G polymorphism and ESCC risk with a relatively large sample size. We found *LEP* rs7799039 A>G was associated with the decreased risk of ESCC in ≥63 years and BMI ≥ 24 kg/m^2^ subgroups. These findings were very similar to the results of previous studies. Hoffsted *et al.* reported that individuals carried the *LEP* rs7799039 AA genotype had higher serum LEP levels than those who carried the *LEP* rs7799039AG or GG genotypes [27]. In this study, we found that *LEP* rs7799039 A>G polymorphism was a protective factor for ESCC, suggesting the presence of the *LEP* rs7799039 G allele, which is associated with the decreased level of LEP, might decrease the risk of ESCC.

Several case-control study focused on the relationship of *LEPR* rs1137101 G>A polymorphism and the risk of cancer. Recently, results of two meta-analyses indicated that this SNP was not associated with the risk of overall cancer [37, 38]. In addition, most of these studies conducted on Caucasian population. The evidence of the association between *LEPR* rs1137101 G>A polymorphism and cancer risk was insufficient in Asians. A previous study suggested that *LEPR* rs1137101 G>A polymorphism might be associated with variation in binding with LEP and, as such, inter-individual differences in serum LEP levels [39]. Just as we mentioned above, LEP may affect cell proliferation, apoptosis, cell migration and angiogenesis. *LEPR* rs1137101 G>A polymorphism may alter the susceptibility of cancer by influencing the ability of binding with LEP. Thus, we aimed to examine the potential association of this polymorphism with the risk of ESCC in Eastern Chinese Han subjects. We found that the *LEPR* rs1137101 G>A polymorphism decreased ESCC risk in ever drinking and ever smoking subgroups. In the future, function of *LEPR* rs1137101 G>A polymorphism should be further explored to confirm these primary findings in ESCC.

Our study had several limitations. Firstly, ESCC patients and controls were enrolled from two hospitals of Jiangsu University and Fujian Medical University and might therefore not be full-representative of the general Eastern Chinese Han population; the possible bias might lead to spurious findings. Secondly, for the limited ESCC patients recruited in this study, this study might have insufficient power to observe the potential relationships. Thirdly, because we only selected some functional polymorphisms in *TCF7L2*, *LEP* and *LEPR* gene, a fine-mapping case-control studies should be conducted in the future. Finally, for lack of some important risk factors, the interactive effect between gene-gene and gene-environment was not further analyzed.

In summary, our findings suggest that *LEPR* rs6588147 G>A polymorphism is associated with the increased risk of ESCC in Eastern Chinese Han population. However, the results of this case-control study highlight that *LEP* rs7799039 A>G and *LEPR* rs1137101 G>A polymorphisms may decrease the risk of ESCC. A fine-mapping study with large sample size and functional exploration is needed to confirm our findings.

## MATERIALS AND METHODS

### Subjects

A total number of 507 ESCC patients and 1,496 non-cancer controls were enrolled in this study. The ESCC patients were from the Affiliated People’s Hospital, Jiangsu University and the Affiliated Union Hospital, Fujian Medical University between August 2013 and December 2016. The diagnosis of ESCC was confirmed based on pathological examination. At the same time, the controls were recruited from physical examination center in these hospitals with sex and age matched. Each subject signed an informed written consent. This study was approved by the Institutional Review Board of Jiangsu University and Fujian Medical University for human subjects (No. SQ20140030, K201408, respectively). When each subject was interviewed, a questionnaire was used to obtain demographic variables and risk factors. And weight and height were also measured. In this study, a BMI ≥ 24 was considered as the criteria for obesity and overweight [40, 41].

### DNA extraction and genotyping

Genomic DNA was carefully isolated from EDTA-anticoagulated blood of recipients by using a Promega DNA blood mini kit (Promega, Madison, USA). *TCF7L2* rs7903146C>T, rs290481 T>C, *LEP* rs7799039 A>G, rs2167270 G>A and *LEPR* rs1137100 G>A, rs1137101 G>A and rs6588147 G>A genotypes were assessed by the SNPscan™ kit (Gnensky Biotechologies Inc., Shanghai, China), which is a double ligation and multiplex fluorescence PCR [42]. For quality control, eighty DNA samples (4%) were randomly selected and genotyped by different colleague. The genotypes of *TCF7L2*, *LEP* and *LEPR* polymorphisms were confirmed.

### Statistical analysis

Continuous variables (e.g. age, height, weight and BMI) are expressed as mean ±standard deviation (SD). Comparisons between ESCC patients and controls were carried out with Student’s t-test. The categorical variables (e.g. *TCF7L2*, *LEP* and *LEPR* genotypes, sex, age, BMI, smoking and drinking status) were compared with Chi-square test (*χ*^2^). Deviations from the HWE for *TCF7L2*, *LEP* and *LEPR* genotypes distribution in controls were evaluated by an internet-based calculator (http://ihg.gsf.de/cgi-bin/hw/hwa1.pl) [43–49]. The relationships of *TCF7L2* rs7903146C>T, rs290481 T>C, *LEP* rs7799039 A>G, rs2167270 G>A and *LEPR* rs1137100 G>A, rs1137101 G>A and rs6588147 G>A polymorphisms with ESCC susceptibility were evaluated by crude ORs and 95% CIs. Multivariate linear regression adjusted for age, sex, BMI, alcohol use and smoking status was used to determine the relationships between *TCF7L2* rs7903146C>T, rs290481 T>C, *LEP* rs7799039 A>G, rs2167270 G>A and *LEPR* rs1137100 G>A, rs1137101 G>A and rs6588147 G>A polymorphisms and ESCC risk with quantitative traits. Data analysis was conducted with SAS software for windows (Version 9.4, SAS Institute, Cary, NC). A *P* < 0.05 (two-tailed) was accepted as the criterion of statistical significance.
